# Cross-sectional study of attitudes about suicide among psychiatrists in Shanghai

**DOI:** 10.1186/1471-244X-14-87

**Published:** 2014-03-25

**Authors:** Yumei Jiao, Michael R Phillips, Yourong Sheng, Guojun Wu, Xianyun Li, Wei Xiong, Liwei Wang

**Affiliations:** 1 Shanghai Mental Health Center, Suicide Research and Prevention Center, Shanghai Jiao Tong University School of Medicine, 3210 Humin Road, Shanghai 201108, China; 2 Department of Psychiatry, Fudan University, Shanghai, China; 3 Departments of Psychiatry and Global Health, Emory University, Atlanta, GA, USA; 4 Shanghai Mental Health Center, Shanghai Jiao Tong University School of Medicine, Shanghai, China; 5 Beijing Huilongguan Hospital, Beijing, China

**Keywords:** Suicide, Attitudes, Psychiatrists, China

## Abstract

**Background:**

Attitudes and knowledge about suicide may influence psychiatrists’ management of suicidal patients but there has been little research about this issue in China.

**Methods:**

We used the Scale of Public Attitudes about Suicide (SPAS) – a 47-item scale developed and validated in China – to assess knowledge about suicide and seven specific attitudes about suicide in a sample of 187 psychiatrists from six psychiatric hospitals in Shanghai. The results were compared to those of 548 urban community members (assessed in a previous study).

**Results:**

Compared to urban community members, psychiatrists were more likely to believe that suicide can be prevented and that suicide is an important social problem but they had more stigmatizing beliefs about suicidal individuals and felt less empathy for them. The belief that suicide can be prevented was more common among female psychiatrists than male psychiatrists but male psychiatrists felt more empathy for suicidal individuals. Only 37% of the psychiatrists correctly agreed that talking about suicide-related issues with an individual would *not* precipitate suicidal behavior and only 41% correctly agreed that those who state that they intend to kill themselves may actually do so.

**Conclusions:**

Many psychiatrists in Shanghai harbor negative attitudes about suicidal individuals and are concerned that directly addressing the issue with patients will increase the risk of suicide. Demographic factors, educational status and work experience are associated with psychiatrists’ attitudes about suicide and, thus, need to be considered when training psychiatrists about suicide prevention.

## Background

Given the high prevalence of mental disorders among individuals who attempt suicide or die by suicide [1-3], the prevention and management of suicidal behavior is often the responsibility of psychiatric professionals, particularly in low- and middle-income countries where the resources for suicide prevention are very limited [4]. Thus, the identification and management of patients with suicidal behavior or ideation is an important part of psychiatric work that should be considered in the assessment of the quality of psychiatric services. But psychiatrists’ attitudes about suicide may influence their willingness and ability to identify and treat patients with suicidal behavior or ideation [5]. Thus, assessing psychiatrists’ attitudes about suicide will provide information that can be used to increase the effectivenss of mental health interventions aimed at reducing suicide [6]. However, despite the potential usefulness of understanding the relationship of psychiatrists’ attitudes about suicide to the effectiveness of suicide prevention activities, there has been little research about psychiatrists’ attitudes about suicide, particularly in low- and middle-income countries [7].

Suicide is an important cause of death in China, particularly among young adults, so the prevention of suicide is an important public health objective for the country. There are, however, few specific programs aimed at achieving this objective [4]. In China, psychiatrists and, to a lesser extent, general physicians are the main professionals responsible for the identification of persons at high risk of suicide, but there has been no attempt to assess their willingness or ability to do this. Moreover, to our knowledge there has never been a study anywhere that compares attitudes about suicide between community members and mental health professionals – an approach that would help identify the socio-cultural context from which psychiatrists’ beliefs about suicide have arisen. As a first step to addressing these issues, in the current study we administered a validated, culture-specific scale that assesses attitudes about suicide to a large sample of psychiatrists working in Shanghai and compared the results to those of urban community members from Tianjin (another large city in China) who were administered the same scale.

## Methods

### Setting

The characteristics of suicide in China are quite different from those reported in other countries – the rates are much higher in rural areas than urban areas, female rates are similar to male rates (in most other countries the male suicide rate is three-fold the female rate), and a lower proportion of suicide decedents have a current mental illness. Despite these differences, the risk factors for suicide are similar to those reported elsewhere [3] and, thus, mental health institutions and mental health professionals need to remain vigilant about the risk of suicidal behavior in their patients.

**Figure 1:**
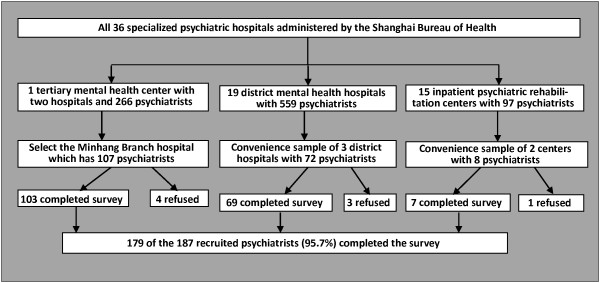
Identification of survey respondents.

The Shanghai municipality, which has a population of 24 million individuals, has a total 39 specialized psychiatric hospitals, 35 of which are administered by the Shanghai Bureau of Health. There are also 3 psychiatric hospitals administered by the Shanghai Bureau of Civil Affairs and 1 hospital administered by the Shanghai Bureau of Public Security. Among the hospitals administered by the Bureau of Health there is one large tertiary center (the Shanghai Mental Health Center, which has two separate hospitals), 19 district psychiatric hospitals (one for each of Shanghai’s 19 districts), and 15 inpatient psychiatric rehabilitation centers in different parts of the city. As shown in Figure 1 there were a total of 922 psychiatrists working in these hospitals at the time of the current study (March and April 2011).

A previous report [8] found that 10.1% (88/869) of inpatients admitted over a one-month period in 2001 to the Shanghai Mental Health Center (SMHC) and 5.5% (79/1430) of inpatients admitted to five of Shanghai’s district-level psychiatric hospitals had a history of prior suicide attempt(s). Another study of patients discharged from the SMHC with a diagnosis of schizophrenia in 2004 [9] found that 3.2% (3/95) had made a suicide attempt and 24.2% (23/95) had suicidal ideation within 18 months of discharge. Thus clinicians at these hospitals have frequent contact with patients who have suicidal ideation or a prior history of suicide attempt.

### Subjects

As shown in Figure 1, the subjects of the current study were psychiatrists working at the Branch Hospital of the SMHC, at three of the district-level psychiatric hospitals (the Shanghai Jing An District Mental Health Center, the Shanghai Fengxian District Mental Health Center, and the Shanghai Minhang District Mental Health Center), and at two of the inpatient psychiatric rehabilitation centers (the Tongning Rehabilitation Center and the Tonghe Rehabilitation Center) from 30 March 2011 to 30 April 2011. The six locations selected for the survey were selected based on ease of access and on the willingness of the hospital leaders to provide administrative support for the study. These six sites had a total of 187 psychiatrists, accounting for 20.3% (187/922) of all psychiatrists working in specialty hospitals administered by the Shanghai Bureau of Health. Among these psychiatrists 179 (95.7%) completed the survey; the other 8 psychiatrists refused to complete the survey.

### Survey instrument

The Scale of Public Attitudes about Suicide (SPAS) [10] was used to assess attitudes about suicide among the participating psychiatrists. This scale was developed in China over a period of three years with the express goal of capturing China-specific attitudes related to suicide. Three waves of focus groups and quantitative surveys with urban community members, rural community members and university students resulted in the final version of the scale [10]. The SPAS includes 47 items; 44 items are divided into seven attitudinal subscales that reflect different attitudes related to suicide and the remaining three items assess basic knowledge about suicide. The attitudes assessed by the seven attitudinal subscales are as follows: (Subscale 1) respondent believes suicide can be prevented, (Subscale 2) respondent believes individuals are able to control their own suicidal tendencies, (Subscale 3) respondent holds stigmatizing attitudes about suicide, (Subscale 4) respondent is understanding of and feels empathy for persons with suicidal behavior, (Subscale 5) respondent believes suicidal behavior is an effective method of controlling others, (Subscale 6) respondent believes that suicide is an important social problem, and (Subscale 7) respondent believes that suicides and suicide attempts are essentially different.

An English-language translation of the scale and a description of how the subscale scores are computed are provided in the Additional file 1. As described in the footnote of the Additional file 1, the seven adjusted subscale scores all range from 0 to 100 points, with higher scores indicating greater agreement with the attitude described in the title of the subscale.

The scale also includes three items (items 10, 20 and 30) about knowledge related to suicide that are relevant in the Chinese context. Qualitative studies conducted during the development of the SPAS [11] found that many community members held incorrect beliefs about suicide: they believed that persons who had attempted suicide would never do it again, that talking about suicide with someone could cause them to more seriously consider taking their lives, and that persons who openly claimed that they intended to take their lives would never actually do it. All three of these ideas are factually incorrect and, if subscribed to by community members or health workers, could seriously undermine the effectiveness of suicide prevention efforts. The three knowledge-related items in the SPAS employ 5-point Likert scales (from ‘definitely agree’ to ‘definitely disagree’) to assess the extent to which respondents agree with the factually correct version of these three ideas: “Persons who have attempted suicide may repeat their suicidal behavior” (item 10); “Talking about suicide-related issues with an individual does *not* precipitate suicidal behavior” (item 20); and “Individuals who say they intend to kill themselves may actually do it” (item 30).

A previous study by Li and colleagues [10] using the SPAS in a representative sample of 548 adults in Tianjin (a large municipality with 14 million residents) reported that the internal consistency and test-retest reliability of six of the seven SPAS subscales were good to excellent (Cronbach’s α and intraclass correlation coefficients [ICC] ranged from 0.62 to 0.87); but Subscale 6 (about the belief that suicide is an important social problem) had relatively poor internal consistency (Cronbach’s α = 0.48) and only fair test-retest reliability (ICC = 0.59).

Detailed demographic and professional information was also obtained for all survey respondents, including gender, age, educational level, professional status, years of working as a psychiatrist, current type of work (inpatient v. outpatient), and type of hospital.

### Data collection

This self-completion survey was adopted as an administrative quality control assessment procedure at the six participating institutions. At each institution an administrator responsible for medical services distributed the survey instrument to all psychiatrists working at the institution, collected the surveys within one week, and verified that all surveys were completed correctly before sending them to the principal investigator for data entry and analysis. The administration of the survey was approved by the academic committee of the Shanghai Mental Health Center and by the medical services departments of all six participating institutions.

### Data analysis

Data were analyzed using SPSS18.0 software. The internal consistency of the seven subscales in the SPAS among these subjects were assessed using Cronbach’s α. The Kolmogorov-Smirnov Z test was used to examine whether or not the results for each of the subscales had a normal distribution. The results from this survey among psychiatrists were compared with the results of a survey of community members in Tianjin Municipality that also used the SPAS [10]. (The raw data from the Tianjin study was available to the authors of the current report.) The univariate comparison of the mean scores for the seven subscales between the two samples used t-tests. These comparisons of the subscale scores between psychiatrists and community members were then repeated after adjusting for differences in gender, age and years of education between the two samples using linear regression methods.

Univariate comparison of mean subscale scores between different subgroups of psychiatrists (e.g., by gender or profession status) were assessed using t-tests and F-tests and the scores were correlated with continuous variables (e.g., age, years of education) using Pearson’s correlation coefficients. Linear regression analyses were used to identify factors independently associated with each of the subscale scores. Gender and age are the factors most strongly correlated with suicide-related behavior, so these variables were controlled for in all of the regression models; however, age and years of working as a psychiatrist were collinear, so only the latter variable was included in the analyses. After forcing gender and years of employment as a psychiatrist into each of the regression models, four other variables (professional status [entered as two dummy variables using attending psychiatrists as the reference group], type of hospital [tertiary hospital v. district hospital or rehabilitation center], years of education, and whether or not the clinician’s current work was limited to inpatient service provision) were entered by a forward stepwise method if significant at the p < 0.05 level.

The three items pertaining to knowledge about suicide were treated as ranked variables (ranging from 1 to 5); comparisons of results for these three items between different groups of subjects and correlation of the item scores with other continuous variables used rank tests (Mann–Whitney U, the Kruskal-Wallis test, and Spearman correlation coefficients). Binary logistic regression models were used to identify demographic factors and attitudes associated with each of these three items. The results for each item were first dichotomized (‘definitely disagree’ , ‘mostly disagree’ and ‘neither agree nor disagree’ were coded as ‘1’; ‘mostly agree’ and ‘definitely agree’ were coded as ‘2’) and the resultant variable was used as the dependent variable in the logistic regression models. Gender and years of working as a psychiatrist were first forced into the models and then eleven other variables (professional status, type of hospital, years of education, current work setting and the seven SPAS subscale scores) were entered by a forward stepwise method if significant at the p < 0.05 level.

## Results

Among the 187 psychiatrists working in the six participating institutions, 179 (95.7%) completed the survey. These respondents included 100 (55.9%) females and 79 (44.1%) males; they had a mean (sd) age of 37.5 (11.3) years, a mean duration of education of 16.1 (1.8) years, and a mean duration of working as psychiatrists of 14.0 (11.7) years; 69 (38.5%) were resident psychiatrists, 82 (45.8%) were attending psychiatrists, and 28 (15.6%) were chief psychiatrists. Compared to the 179 who completed the survey, the 8 who refused to participate were somewhat older (45.9 [16.2] years v. 37.5 [11.3] years, t = 2.00, p = 0.047) and more of them were chief psychiatrists (1 [12.5%] was a resident psychiatrist, 3 [37.5%] were attending psychiatrists, and 4 [50.0%] were chief psychiatrists; X^2^ = 6.20, df = 2, p = 0.045).

### Psychometric characteristics of the SPAS scale in these respondents

As shown in Table 1, the Cronbach α values of the seven SPAS subscales ranged from 0.59 to 0.81, which indicates fair to excellent internal consistency. Moreover, based on the Kolmogorov-Smirnov test, the distributions of the seven subscale scores were not significantly different from normal. The correlation of the seven subscale scores with each other (using Pearson correlation coefficients) ranged from −0.38 to 0.47; the mean absolute value of the 21 correlation coefficients (for the 21 different pairs of subscales) was 0.24.

**Table 1:** Internal validity (alpha) of subscale scores of the Scale of Public Attitudes about Suicide (SPAS) among psychiatrists in Shanghai and comparison of attitudes about suicide between psychiatrists in Shanghai and urban community members with and without adjustment for gender, age and years of education

**SPAS subscales**	**Number of items**	**Alpha**	**Normality**		**Comparison of mean (sd) subscale scores without adjustment**				**Comparison adjusted for age, gender and years of education using linear regression models**			
			**Kolmogorov -Smirnov Z**^**a**^	**p**	**Psychiatrists [n = 179] mean (sd)**	**Community members**^**b **^**[n = 548] mean (sd)**	***t*-test**	**p**	**β**	**Beta**	**95% CI of β**	**p**
**Subscale 1**^**c**^	6	0.586	1.28	0.075	69.2 (15.4)	47.0 (21.5)	15.05	<0.001	31.2	0.60	27.1 ~ 35.4	<0.001
**Subscale 2**^**c**^	5	0.619	1.32	0.063	63.7 (16.1)	51.0 (22.0)	8.31	<0.001	14.6	0.29	10.2 ~ 19.1	<0.001
**Subscale 3**^**c**^	10	0.762	0.97	0.308	45.3 (14.5)	33.4 (21.2)	8.43	<0.001	5.6	0.12	1.5 ~ 9.7	0.007
**Subscale 4**^**c**^	7	0.715	1.03	0.242	41.0 (16.9)	60.9 (20.8)	−12.92	<0.001	−17.8	−0.35	−22.1 ~ −13.5	<0.001
**Subscale 5**^**c**^	6	0.622	1.13	0.154	42.8 (15.8)	52.1 (20.6)	−6.26	<0.001	−12.9	−0.28	−17.0 ~ −8.7	<0.001
**Subscale 6**^**c**^	5	0.678	1.12	0.163	71.0 (16.0)	38.4 (19.5)	22.35	<0.001	29.6	0.54	25.6 ~ 33.5	<0.001
**Subscale 7**^**c**^	5	0.814	1.01	0.265	57.0 (21.5)	62.6 (22.9)	−2.88	0.004	−6.1	−0.12	−10.9 ~ −1.3	0.013

^a^The Kolmogorov-Smirnov test is an overall assessment of normality (including consideration of kurtosis and skewedness); if the p-value is >0.05 the distribution is considered normal.

^b^Results provided in 2011 study by Li and colleagues.

^**c**^Adjusted total scores for all seven attitudinal subscales have a range of 0–100.

Subscale 1: Respondent believes suicide can be prevented.

Subscale 2: Respondent believes individuals are able to control their own suicidal tendencies.

Subscale 3: Respondent holds stigmatizing attitudes about suicide.

Subscale 4: Respondent is understanding of and feels empathy for persons with suicidal behavior.

Subscale 5: Respondent believes suicidal behavior is an effective method of controlling others.

Subscale 6: Respondent believes that suicide is an important social problem.

Subscale 7: Respondent believes that suicides and suicide attempts are essentially different.

### Comparison of attitudes of psychiatrists with that of urban community members

As shown in Table 1, all seven attitudes about suicide assessed by the SPAS among psychiatrists in Shanghai were significantly different from those in adult community members in Tianjin. Moreover, these differences remained statistically significant after adjusting for age, gender and years of education. Psychiatrists were more likely than community members to believe that suicide can be prevented, that individuals are able to control their suicidal tendencies, that suicide is an important social problem, and that fatal and non-fatal suicide are quite similar; but they were less likely to believe that suicidal behavior is an effective method of controlling others. Somewhat surprisingly, psychiatrists had more stigmatizing beliefs about suicidal individuals and felt less empathy for suicidal individuals.

### Relationship between demographic factors and SPAS subscales scores

Table 2 shows the univariate relationships between each of the SPAS subscale scores and the characteristics of the 179 respondents. Compared to male psychiatrists, female psychiatrists were more likely to believe that suicide is preventable but less likely to feel empathy for individuals with suicidal behavior. Psychiatrists who have a longer mean duration of formal education are less likely to have stigmatizing attitudes about suicide and more likely to feel empathy for persons with suicidal behavior; however, there were no corresponding differences based on the educational degree (i.e., technical college, bachelor’s degree, or master’s degree) attained by the psychiatrists. Compared to attending psychiatrists and chief psychiatrists, resident psychiatrists were more likely to consider fatal and non-fatal suicidal behavior as essentially different; this was also the case for younger psychiatrists and for psychiatrists who only work on inpatient settings (most of whom are resident psychiatrists). Compared to psychiatrists working in the tertiary psychiatric center, psychiatrists working in the lower-level institutions (i.e., district hospitals and rehabilitation centers) had more stigmatizing attitudes about suicide and were more likely to believe that suicide is an important social problem.

**Table 2:** Relationship of sociodemographic variables and the seven attitudes about suicide assessed by the subscales of the Scale of Public Attitudes about Suicide (SPAS) among 179 psychiatrists from Shanghai

**Variable**	**n**	**Subscale 1**^**a**^	**Subscale 2**^**a**^	**Subscale 3**^**a**^	**Subscale 4**^**a**^	**Subscale 5**^**a**^	**Subscale 6**^**a**^	**Subscale 7**^**a**^
**Age**								
r	179	−0.08	−0.12	0.11	−0.00	−0.00	0.04	*−0.32*
p		0.271	0.108	0.142	0.978	0.986	0.556	*<0.001*
**Years working as a psychiatrist**								
r	179	−0.03	−0.11	0.06	0.01	−0.02	0.06	*−0.28*
p		0.692	0.144	0.467	0.925	0.785	0.509	*<0.001*
**Years of education**								
r	179	0.03	0.06	*−0.20*	*0.18*	0.11	−0.02	0.08
p		0.716	0.452	*0.007*	*0.017*	0.134	0.803	0.282
		***mean (sd)***	***mean (sd)***	***mean (sd)***	***mean (sd)***	***mean (sd)***	***mean (sd)***	***mean (sd)***
**Gender**								
Male	79	*66.2 (15.8)*	61.2 (15.9)	44.1 (15.8)	*46.1 (17.7)*	45.4 (14.9)	71.5 (15.4)	53.9 (21.4)
Female	100	*71.5 (14.7)*	65.6 (16.1)	46.3 (13.5)	*36.9 (15.1)*	40.8 (16.3)	70.6 (16.6)	59.4 (21.4)
*t*-test		*−2.29*	−1.83	−1.01	*3.73*	1.94	0.38	1.72
p		*0.023*	0.069	0.313	*<0.001*	0.054	0.705	0.088
**Professional status**								
Resident psychiatrist	69	69.6 (16.3)	64.5 (16.9)	43.7 (12.3)	42.4 (14.6)	44.0 (16.4)	70.2 (14.0)	*63.1 (19.0)*
Attending psychiatrist	82	69.3 (15.2)	63.0 (15.9)	47.6 (16.2)	39.3 (18.2)	41.8 (15.3)	70.8 (17.8)	*54.1 (21.9)*
Chief psychiatrist	28	67.9 (14.0)	63.6 (14.9)	42.5 (14.0)	42.3 (18.0)	43.3 (15.9)	73.4 (15.8)	*50.4 (23.0)*
F-test		0.13	0.16	2.03	0.75	0.37	0.40	*5.08*
p		0.881	0.850	0.134	0.476	0.690	0.675	*0.007*^*b*^
**Type of degree**								
Technical college	29	68.1 (15.0)	61.7 (14.8)	50.6 (16.2)	37.4 (16.0)	42.8 (16.6 )	74.5 (14.4)	50.3 (23.9)
Bachelor’s degree	122	69.6 (15.8)	63.9 (16.6)	44.8 (14.3)	40.5 (17.2)	41.4 (15.2 )	70.2 (16.6)	58.2 (21.5)
Master’s degree	28	68.4.(14.5)	64.6 (15.3)	42.2 (12.7)	46.7 (15.4)	49.4 (16.4)	71.1 (15.3)	58.9 (18.3)
F-test		0.15	0.27	2.68	2.33	3.02	0.85	1.69
p		0.863	0.762	0.071	0.101	0.052	0.430	0.187
**Type of hospital**								
District hospital or inpatient rehabilitation center	76	68.7 (16.1)	62.6 (15.1)	*48.8 (15.3)*	38.5 (16.4)	40.7 (16.5)	*75.1 (15.0)*	55.7 (23.0)
Tertiary psychiatric center	103	69.5 (14.9)	64.5 (16.8)	*42.8 (13.5)*	42.8 (17.0)	44.4 (15.1)	*68.0 (16.2)*	58.0 (20.4)
*t*-test		−0.36	−0.78	*2.79*	−1.70	−1.55	*3.02*	−0.69
p		0.718	0.436	*0.006*	0.090	0.124	*0.003*	0.493
**Currently only treats inpatients**								
Yes	79	70.1 (15.7)	64.6 (17.2)	45.0 (13.1)	41.5 (16.5)	43.5 (17.1)	71.3 (15.0)	*61.1 (20.6)*
No	100	68.5 (15.2)	63.0 (15.2)	45.5 (15.6)	40.6 (17.2)	42.3 (14.8)	70.8 (16.9)	*53.7 (21.7)*
*t*-test		0.71	0.66	−0.24	0.35	0.49	0.19	*2.31*
p		0.482	0.509	0.810	0.729	0.627	0.848	*0.022*

^a^See footnote in Table 1 for description of the attitudes considered by each of the seven subscales.

^b^multiple comparison test result: level in resident psychiatrists was significantly higher than in attending psychiatrists and chief psychiatrists.

The multivariate results about the factors independently associated with each of the SPAS subscale scores are shown in Table 3. After forcing gender and years of working as a psychiatrist into the seven linear regression models, the four other characteristics considered (type of hospital, professional status, years of education, and current work setting) were entered if they were significant at the p = 0.05 level. The results show that compared to female psychiatrists male psychiatrists were less likely to believe that suicide can be prevented, more likely to believe that suicidal behavior is an effective method of controlling others, and more likely to feel empathy for persons with suicidal behavior. Those who had worked longer as a psychiatrist were more likely to believe that fatal and non-fatal suicidal behaviors were similar. Compared to psychiatrists from the lower-level institutions, psychiatrists from the tertiary psychiatric center were less likely to consider suicide an important social problem and less likely to hold stigmatizing attitudes about suicide. And psychiatrists with more formal education were more likely to feel empathy for persons with suicidal behavior.

**Table 3:** **Results of seven linear regression analyses of factors associated with the seven attitudes about suicide assessed by the subscales of the Scale of Public Attitudes about Suicide (SPAS) in 179 psychiatrists from Shanghai**^**a**^

**Attitudes**	**β**	**Beta**	**95% CI of β**	**p**
***Subscale 1: Respondent believes suicide can be prevented (0–100)***				
Male	*−5.38*	*−0.17*	*−10.07 ~ −0.69*	*0.025*
Years working as a psychiatrist	0.02	0.02	−0.18 ~ 0.22	0.843
***Subscale 2: Respondent believes individuals are able to control their own suicidal tendencies***				
Male	−3.73	−0.12	−8.65 **~** 1.19	0.136
Years working as a psychiatrist	−0.11	−0.08	−0.32 **~** 0.10	0.303
***Subscale 3: Respondent holds stigmatizing attitudes about suicide***				
Male	−2.91	−0.10	−7.31 ~ 1.49	0.193
Years working as a psychiatrist	0.06	0.05	−0.13 ~ 0.25	0.538
Psychiatrist from tertiary psychiatric center	*−5.95*	*−0.20*	*−10.28 ~ −1.63*	*0.007*
***Subscale 4: Respondent is understanding of and feels empathy for persons with suicidal behavior***				
Male	*9.43*	*0.28*	*4.50 ~ 14.36*	*<0.001*
Years working as a psychiatrist	0.06	0.04	−0.18 ~ 0.29	0.638
Years of education	*2.13*	*0.22*	*0.59 ~ 3.66*	*0.007*
***Subscale 5: Respondent believes suicidal behavior is an effective method of controlling others***				
Male	*5.09*	*0.16*	*0.26 ~ 9.92*	*0.039*
Years working as a psychiatrist	−0.08	−0.06	−0.29 ~ 0.12	0.422
***Subscale 6: Respondent believes that suicide is an important social problem***				
Male	0.34	0.01	−4.51 ~ 5.18	0.892
Years working as a psychiatrist	0.03	0.02	−0.18 ~ 0.24	0.766
Psychiatrist from tertiary psychiatric center	*−7.03*	*−0.22*	*−11.80 ~ −2.26*	*0.004*
***Subscale 7: Respondent believes that suicides and suicide attempts are essentially different***				
Male	−2.63	−0.06	−9.01 ~ 3.75	0.418
Years working as a psychiatrist	*−0.48*	*−0.26*	*−0.75 ~ −0.21*	*0.001*

^a^The seven attitudes are assessed on continuous scales with a range of 0 to 100. In all seven analyses two variables were initially forced into the model (gender, and years of working as a psychiatrist) and then four other variables (type of hospital [tertiary psychiatric center v. district hospital or rehabilitation center], professional status [entered as two dummy variables using attending psychiatrist as the reference group], years of education, and whether or not the clinician’s current work was limited to inpatient service provision) were entered by a forward stepwise method if significant at the p < 0.05 level.

### Relationship between knowledge about suicide, respondent characteristics, and respondent attitudes

As show in Table 4, 91% of the 179 psychiatrists and 73% of the 548 community respondents agreed that persons who have attempted suicide may have subsequent suicidal behavior which, of course, is the correct response. On the other hand, only 37% of the psychiatrists and 13% of community respondents correctly agreed that talking about suicide-related issues with an individual would *not* precipitate suicidal behavior and only 41% of the psychiatrists and 25% of community respondents correctly agreed that those who state that they intend to kill themselves may actually do it. The proportion of correct responses (i.e., ‘definitely agree’ or ‘mostly agree’ to three statements in Table 4) for each of the three questions among psychiatrists was significantly higher than in the community respondents and these differences remained statistically significant after adjusting for age, gender, and years of education in a logistic regression analysis.

**Table 4:** Responses to three questions about suicide-related knowledge from the Scale of Public Attitudes about Suicide (SPAS) by 179 psychiatrists from Shanghai and 548 urban community members

**Question**	**Sample**	**Definitely agree%**	**Mostly agree%**	**Neither agree nor disagree%**	**Mostly disagree%**	**Definitely disagree%**
Persons who have attempted suicide may repeat their suicidal behavior^a^	Psychiatrists	50.3	40.8	7.3	1.7	0.0
	Community members	36.3	36.3	12.2	6.8	8.4
Talking about suicide-related issues with an individual does *not* precipitate suicidal behavior^b^	Psychiatrists	12.3	24.6	25.7	29.6	7.8
	Community members	5.5	7.2	13.7	28.3	45.3
Individuals who say they intend to kill themselves may actually do it^c^	Psychiatrists	15.6	25.7	21.2	32.4	5.0
	Community members	10.2	14.6	17.7	38.1	19.3

^a^Mann–Whitney rank test Z = 4.90 (p < 0.001).

^b^Mann–Whitney rank test Z = 10.12 (p < 0.001).

^c^Mann–Whitney rank test Z = 5.43 (p < 0.001).

In the univariate analysis, responses to these three knowledge-related items did not differ significantly by gender, age, years of working as a psychiatrist, professional status, type of hospital, or work setting. However, respondents with more years of formal education were more likely to agree that individuals who say they intend to kill themselves may actually do it (*r*_*s*_ = 0.17. *p* = 0.027). Responses to these three items about suicide-related knowledge were significantly correlated with several of the SPAS subscale scores, though these correlations were weak to moderate in strength; the absolute value of the statistically significant Spearman’s correlation coefficients ranged for 0.17 to 0.33. (Results provided on request.)

Results of the binary logistic regression analysis to identify factors independently related to these three knowledge items are shown in Table 5. Psychiatrists working in the tertiary center and those who believe that suicide is an important social problem were more likely to agree that persons who have attempted suicide are more likely to have subsequent suicidal behavior. Psychiatrists who believe that suicide is an important social problem and those who believe that suicide and attempted suicide are essentially different were more likely to agree that talking about suicide with an individual does not precipitate suicidal behavior. And psychiatrists with a longer formal education, those who believe that suicide is an important social problem, those who believe that suicide can be prevented, and those who believe that suicide is an effective method of controlling others were more likely to agree that individuals who say they intend to kill themselves may actually do it.

**Table 5:** **Results of logistic regression analysis of factors associated with responses to three questions about suicide-related knowledge from the Scale of Public Attitudes about Suicide (SPAS) by 179 psychiatrists in Shanghai**^**a**^

***SPAS items relating to knowledge about suicide***	**Wald**	**p**	**Odds Ratio (OR)**	**95% CI of OR**
***Persons who have attempted suicide may repeat their suicidal behavior***				
Male	0.75	0.386	1.76	0.49 ~ 6.28
Years of experience in psychiatry	0.25	0.620	0.99	0.92 ~ 1.05
Subscale 6: Believes that suicide is an important social problem	*11.12*	*<0.001*	*1.09*	*1.04 ~ 1.14*
Psychiatrist from tertiary psychiatric center	*7.74*	*0.005*	*7.45*	*1.81 ~ 30.67*
***Talking about suicide-related issues with an individual does not precipitate suicidal behavior***				
Male	0.03	0.864	1.06	0.55 ~ 2.04
Years of experience in psychiatry	0.79	0.376	0.99	0.96 ~ 1.02
Subscale 6: Believes that suicide is an important social problem	*6.45*	*0.011*	*1.03*	*1.01 ~ 1.05*
Subscale 7: Believes that suicide and suicide attempt are essentially different	*4.53*	*0.033*	*0.98*	*0.97 ~ 1.00*
***Individuals who say they intend to kill themselves may actually do it***				
Male	2.65	0.104	1.77	0.89 ~ 3.52
Years of experience in psychiatry	0.46	0.500	1.01	0.98 ~ 1.04
Years of education	*3.89*	*0.049*	*1.25*	*1.00 ~ 1.57*
Subscale 5: Believes that suicide is an effective method of controlling others	*6.38*	*0.012*	*1.03*	*1.01 ~ 1.05*
Subscale 6: Believes that suicide is an important social problem	*6.16*	*0.013*	*1.03*	*1.01 ~ 1.05*
Subscale 1: Believes that suicide can be prevented	*6.02*	*0.014*	*1.03*	*1.01 ~ 1.06*

^a^In the three logistic regression analyses, responses to the three variables about suicide knowledge were dichotomized: ‘definitely disagree’ , ‘mostly disagree’ and ‘neither agree nor disagree’ were coded as ‘1’; ‘mostly agree’ and ‘definitely agree’ were coded as ‘2’. Two variables were initially forced into the models (gender, and years of working as a psychiatrist) and then eleven other variables (type of hospital [tertiary psychiatric center v. district hospital or rehabilitation center], professional status [entered as two dummy variables using attending psychiatrist as the reference group], years of education, whether or not the clinician’s current work was limited to inpatient service provision and the seven subscales scores of the SPAS) were entered by a forward stepwise method if significant at the p < 0.05 level.

## Discussion

### Main findings

To our knowledge this is the first study on the attitudes about suicide among mental health professionals in China and the first study anywhere to directly compare the attitudes about suicide between community members and mental health professionals. The instrument used to assess these attitudes – the Scale of Public Attitudes about Suicide (SPAS) – was developed in China over a number of years and covers seven types of attitudes, several of which have not been considered in studies on attitudes from other countries. The psychometric properties of the scale are acceptable both when administered to community members and to psychiatrists. The sample of psychiatrists who completed the survey was relatively large and included psychiatrists from different types of psychiatric hospitals.

The study found that attitudes about suicide among psychiatrists were significantly different from those of community members for all seven attitudes assessed by the SPAS. As expected, psychiatrists were more likely than community members to believe that suicide is an important social problem and that suicide can be prevented but, unexpectedly, they were also more likely to hold stigmatizing beliefs about suicidal individuals and had less understanding of and sympathy for these individuals. These differences in attitudes remained statistically significant after adjusting for gender, age and level of education so the demographic characteristics of psychiatrists do not explain these major attitudinal differences between psychiatrists and community members. Some of these attitudinal differences may be true of all types of health professionals, so further studies that administer the SPAS to non-psychiatric clinicians would be needed to determine the extent to which these differences in attitudes about suicide are specific to psychiatric clinicians. Some studies from other countries that compare the attitudes and knowledge about suicide between mental health professionals and other types of health professionals report that mental health professionals have less negative attitudes [12-14], but this has not, as yet, been done in China

The stigmatizing attitudes held by psychiatrists about suicidal individuals and their relative lack of sympathy for these individuals could be related to the stresses and risks psychiatrists face when treating suicidal patients. When a patient dies of suicide it is an enormous professional and legal burden for both the psychiatrist and for the psychiatrist’s institution. This is particularly true in the United States where the suicidal death of a patient is the most common cause of complaints about psychiatric negligence [15,16], and where a large proportion of insurance claims for psychiatric malpractice are compensation for suicides [16,17]. This issue of litigation related to suicide has become progressively more important in the clinical practice of psychaitry in China over the last 15 years [18], so it is not surprising that psychiatrists are increasingly anxious about treating acutely suicidal patients and, thus, harbour negative attitudes about such patients.

Most researchers in China [19] and elsewhere [14,20] report that attitudes about suicide are related to cultural traditions, religious beliefs, occupation and other demographic factors. In support of these previous findings, the current study also found differences in attitudes about suicide between different subgroups of psychiatrists. For example, female psychiatrists showed *less* empathy for individuals with suicidal behavior than male psychiatrists. This result is different from that of studies from high-income countries [21] but it parallels the findings of a study among health workers in general hospital settings in Beijing [22] which found that male staff members were more accepting and understanding of suicidal behavior than female staff members. However, another study among a mixed sample of students and health workers in Suzhou, China [23] found no differences in attitudes by gender. Our study also found that psychiatrists from low-level institutions (district psychiatric hospitals and community rehabilitation centers) were more likely than psychiatrists from a tertiary psychiatric center to hold discriminatory beliefs about suicidal individuals and to believe that suicide was an important social problem. This result confirms the findings of studies from other countries [24] which report that the clinical setting is one of the factors that influences clinicians’ willingness to help suicidal patients. Thus interventions aimed at training psychiatric professionals about suicide prevention may need to be tailor-made for the type of institution and for the type of health professional being trained.

Previous studies in other countries [25] have reported that clinicians’ attitudes and knowledge about suicide are directly related to the identification and prevention of suicidal behavior. In our study only 37% of the psychiatrists who participated in the survey agreed that talking about suicide with patients did not increase the risk of subsequent suicidal behavior, and only 41% agreed that individuals who clearly state that they intend to kill themselves may actually do so. Thus the majority of psychiatrists held incorrect views about the risk of suicide in these two situations. These incorrect beliefs were also common in community respondents so it would appear that education as a physician and as a psychiatrist in China does little to change these beliefs. Clearly, efforts need to be made to correct this misinformation in psychiatrists, in other health professionals and, ultimately, in the public at large. But we also found that these incorrect views about the risk of suicide are more common in psychiatrists who did not believe that suicide was an important social issue. Thus efforts to improve clinicians’ management of suicidal individuals must both change their knowledge about suicide and, more importantly, change their attitudes about suicide and suicidal individuals.

### Limitations

A number of limitations should be considered when interpreting these results. The internal consistency measures of the subscales of the SPAS as assessed in this sample of psychiatrists were satisfactory, but we did not assess the test-retest reliability of the scale so we cannot be certain of the stability of these attitudes over time among psychiatrists. The completion rate for the survey was quite high (96%) so the results are representative of all psychiatrists working in the six participating hospitals, but these institutions were not randomly selected from all psychiatric institutions in Shanghai so the sample may not be fully representative of all psychiatrists in the municipality, or of psychiatrists in other parts of the country. The community sample used for comparison with the Shanghai psychiatrists was collected in Tianjin—another large city in China; the Tianjin results may not be the same as those for community residents in Shanghai, though we expect any differences between community attitudes in Tianjin and Shanghai to be minor. Questionnaires were distributed to respondents and then returned a couple of days later; individuals were instructed to independently complete the surveys but it is not possible to be certain that they did so. The number of demographic variables collected on respondents was limited so it is possible that some important factors (e.g., family history of suicidal behavior) were not identified. Finally, only three specific knowledge-related items are considered in the SPAS –those that assessed incorrect beliefs prevalent in the Chinese community—so this scale does not provide a comprehensive assessment of respondents’ knowledge about suicide.

## Conclusions

There are two major findings from the study that need to be confirmed in other locations in China and, possibly, in other countries. (1) Psychiatrists’ attitudes about suicide are significantly different from those of community members. Notably, psychiatrists have more stigmatizing attitudes about suicide than community members and, surprisingly, felt less empathy for suicidal individuals. (2) The majority of psychiatrists concurred with the incorrect community belief that talking about suicide with an individual would increase the risk of subsequent suicidal behavior in the individual.

Do these beliefs affect the way psychiatrists interact with suicidal individuals and, of even greater concern, influence their willingness to regularly ask patients about suicidal ideation or behavior? It certainly seems possible that this is the case, but further work is needed to confirm the expected connection between the attitudes of psychiatrists and their clinical behavior. If there is a causal link between these attitudes and clinical practices, targeted intervention studies aimed at changing the attitudes of psychiatrists will be needed as a first step to improve their ability to prevent suicidal behavior.

## Abbreviations

SMHC: Shanghai Mental Health Center; SPAS: Scale of Public Attitudes about Suicide.

## Competing interests

The authors report no competing interest related to this manuscript. This study was sponsored by the Shanghai Mental Health Center Pilot Study Fund (Project Number: 2011-YJ-08) and by the National Natural Science Foundation of China (NSFC, No. 81371502). The funders did not play any role in the design, conduct, analysis or write-up of the study.

## Authors’ contributions

YJ designed the study, coordinated the data collection, conducted the analysis and prepared the initial draft of the paper. MP designed the survey instrument, assisted in the analysis of the data and edited the paper. YS and GW helped coordinate the study at the six participating hospitals. XL assisted in the analysis of the data from the comparison community sample. WX assisted in the data analysis and in the write-up of the results. LW participated in the conceptualization of the study and in the interpretation of the results. All authors read and approved the final manuscript.

## Pre-publication history

The pre-publication history for this paper can be accessed here:

http://www.biomedcentral.com/1471-244X/14/87/prepub

## Supplementary Material

Additional file 1 **Appendix 1.** Scale of Public Attitudes about Suicide (SPAS) (translated from the original Chinese). Click here for file
