# Supplementary material for: Cross-sectional study of attitudes about suicide among psychiatrists in Shanghai
Source: BMC Psychiatry. 2014 Mar 25;14:87. doi: 10.1186/1471-244X-14-87 (PMC3987172; doi:10.1186/1471-244X-14-87)
Supplement: Additional file 1 — Appendix 1. Scale of Public Attitudes about Suicide (SPAS) (translated from the original Chinese). [file 1471-244X-14-87-S1.pdf]

Appendix

Scale of Public Attitudes about Suicide (SPAS) (translated from the original Chinese)	
**Item sequence**	***Subscale 1: Respondent believes suicide can be prevented*** ***[Note: Items in this subscale are reverse coded; higher scores indicate disagreement with the content of the item]***
4	Suicidal behavior is not predictable
14	In most situations, individuals and organizations should not intervene when a person is suicidal
24	Public mental health education does not help to decrease suicide rates
34	Suicide can't be prevented
37	Better management of the means of suicide such as pesticides and lethal drugs will not reduce the occurrence of suicidal behavior
43	Once someone decides to take their life, it's hard to change their mind
	Subscale 2: Respondent believes individuals are able to control their own suicidal tendencies ***[Note: Items in this subscale are reverse coded; higher scores indicate disagreement with the content of the item]***
5	Suicide occurs when an individual feels that there is no other choice
15	The factors that lead up to a suicide are beyond the control of the individual
25	Suicidal individuals are not responsible for taking the path that leads to suicide
35	Suicide is not due to the faults of the individual
44	Suicide is an act that is beyond the power of the individual to control
	Subscale 3: Respondent holds stigmatizing attitudes about suicide
2	Suicide is an act that betrays family and friends
6	Everyone looks down on the behavior of people who take their own lives
11	People are infuriated by suicidal behavior
16	Most people despise individuals who have suicidal behavior
21	A suicidal act is unquestionably an irresponsible behavior
26	People who die by suicide or attempt suicide are stupid
31	Suicidal behavior occurs in persons with low self-esteem
36	People with suicidal behavior are poorly educated
41	People with suicidal behavior are usually selfish
47	Suicide is an act of extreme cowardice
	Subscale 4: Respondent is understanding of and feels empathy for persons with suicidal behavior
1	In some circumstances suicide is a responsible act
7	Persons who are suicidal deserve our sympathy
12	Sometimes suicide is a courageous act
17	Taking one's own life to benefit others is commendable
22	Most people pity those who die by suicide or attempt suicide
32	Sometimes suicide is a rational act
40	In some situations suicidal behavior is worthy of respect
	Subscale 5: Respondent believes suicidal behavior is an effective method of controlling others
8	People can use their death by suicide as a method to achieve other goals
13	The goal of most elders with suicidal behavior is to decrease the burden on the family
18	Most suicidal acts are primarily aimed at influencing others
28	The main goal of suicidal acts precipitated by interpersonal conflicts is to punish or threaten others
38	Making suicide threats is a way to alter the behavior of relatives and friends
45	Persons who attempt suicide can use their suicidal behavior to achieve personal goals

Scale of Public Attitudes about Suicide (SPAS) (continued)	
**Item sequence**	***Subscale 6: Respondent believes that suicide is an important social problem***
9	Suicidal behavior causes substantial economic loss for the country
23	Suicidal behaviors often have negative effects on social stability
29	Suicide in the society negatively affects the healthy development of all adolescents
39	Suicide is a social problem that deserves the expenditure of substantial resources
46	Suicidal behavior has serious effects on the subsequent lives of family members
	Subscale 7: Respondent believes that suicides and suicide attempts are essentially different ***[Note: Items in this subscale are reverse coded; higher scores indicate disagreement with the content of the item]***
3	Persons who die by suicide and those who attempt suicide share the same personality traits
19	The severity of the life stressors experienced by persons who die by suicide is similar to that of persons who attempt suicide
27	The intent to die at the time of the suicidal act in persons who attempt suicide is as strong as that in those who die by suicide
33	The underlying causes of suicidal acts are the same in persons who die by suicide as the causes in persons who attempt suicide
42	The goals of the suicidal act in persons who die by suicide are similar to the goals of persons who attempt suicide
	Three questions about suicide-related knowledge
10	Persons who have attempted suicide may repeat their suicidal behavior
20	Talking about suicide-related issues with an individual does not precipitate suicidal behavior
30	People who say they intend to kill themselves may actually do it

Respondents to the 47 items in this self-completion survey (which are presented in the sequence shown in the 'item sequence' column) select the response that most closely represents their degree of agreement with the statement from five choices: 'definitely agree,' 'mostly agree,' 'neither agree nor disagree,' 'mostly disagree,' and 'definitely disagree.' In subscales 3,4,5, and 6 responses are scored from 5 to 1 (i.e., definitely agree=5 to definitely disagree=1); reverse scoring is used for subscales 1, 2, and 7 (i.e., definitely agree=1 to definitely disagree=5). The raw subscale scores are the sum of the item scores for the items in the subscales; these raw scores are converted to adjusted scores on a 0 to 100 scale using the following formula: (100 * [raw subscale score - number of items in subscale]/[4 * number of items in subscale]). The higher the adjusted subscale score, the more strongly the respondent agrees with the attitude described in the name of the subscale.
